# A Royal Appeal

**Published:** 1920-05-22

**Authors:** 


### The Hospital

The Workers' Newspaper of Administrative Medicine and Institutional Life, Administration, National Insurance and Health.

No. 1772, Vol. LXVIII. SATURDAY, MAY 22, 1920. PRICE TWOPENCE

### A ROYAL APPEAL.

Her Majesty Queen Alexandra and H.R.H. the Duke of Connaught have issued a signed appeal on behalf of the peace work of the Joint Council of the British Red Cross Society and the Order of S. John of Jerusalem in England (see p. 199). This document is one of the very few of which it can be said that the signature is more important than the text. For what does the document thus signed expressly indicate? It means that, at a time of severe financial difficulty, Her Majesty Queen Alexandra has lent her Royal support to the cause of voluntary hospitals, with the result that any who waver in their allegiance to the voluntary system are placed in the disagreeable position of seeming to decline Her Majesty's lead.

The text of the appeal is worthy of its signature. For, after pointing out the work arising from the war, still unfinished in regard to those who are still on active service, the needs of the demobilised, of the orthopædic clinics, of the Home Service ambulance organisation, and of the "War and Peace" Library, the appeal expressly mentions the following immediate objects:—"Assistance, financial and otherwise, to the Voluntary Civil Hospitals, in view of the strain put upon these hospitals by the war," and "assistance where required in all branches of nursing. . . ." This phrase should remind the public that, while it is as President of the British Red Cross Society that Her Majesty has signed this appeal, Queen Alexandra is also President of the Royal National Pension Fund for Nurses. The voluntary principle in general, the welfare of the nurse in particular, have always been honoured by her peculiar regard. Now the mainstay of the voluntary system is a certain article of faith, namely, that personal service in the cause of the sick is the best thanksgiving for health. This point, which second-rate minds dislike to see kept in the foreground, is emphasised by Her Majesty in expressing the hope of the signatories that those who, in peace time, are in suffering or distress will feel, when helped by the Order of S. John and the British Red Cross Society, that they are receiving only the just and proper tribute, from those who are enjoying health and strength, to their fellow men and women who are less fortunate than themselves.

The appeal contains an interesting extract from Article XXV. of the Covenant of the League of Nations, under which the Joint Council possesses general power to act in all matters concerned with "the improvement of health, the prevention of disease, and the mitigation of suffering throughout the world." Consequently, the whole field of that which we may call hospital activity has been surveyed, and the funds derived from the appeal will be allotted generally to preventive, research, therapeutic, and nursing work. From the needs of tuberculosis to those of child welfare the divisions of the health service are marked for support. It is probably the most comprehensive attempt ever made to maintain a national health service on voluntary lines and by means of voluntary contributions. When we consider the signature under which this appeal is issued, the financial difficulties of the time, and the immense prestige which the Joint Societies, under the leadership of Sir Arthur Stanley, gained during the war, the failure of the appeal is improbable. In every sense of the word it is well timed. Reconstruction ceasing merely to be a hope has become, in hospital affairs, a reality. For not the least eloquent part of the appeal is the tabular statement which embodies the findings of Sir Napier Burnett's survey. That this may be studied with the care which it deserves, we publish only a digest of the survey in the present number. The first thing to do is to meditate upon Queen Alexandra's message and, when that shall have been appreciated, to study the situation which occasions it. The secret of all voluntary finance is to begin with idealism and to end with statistics.

The old question "what is the cost per occupied bed" at present cannot be answered satisfactorily.

The time is now to place all hospital statistics on a proper basis by a common method of appraisement. This Sir Napier Burnett's survey should help to bring about. The reasons for the increase in surgical cases is one worth study, for it shows, not that medical cases are disappearing, but that people have learned not to have operations performed in their own homes. The time will come, so long as the voluntary system remains to humanise the hospitals, when the public will have resort to hospitals also for the treatment of such complaints as heart or kidney troubles. Medical wards will come into their own again, perhaps in a separated block, or a country home of the main institution.

A scheme for the future assistance of the voluntary civil hospital includes not only a plan for raising the sum of one million annually, which we do not believe Sir Arthur Stanley will find very difficult, but a plea for co-operative buying, and the coordination of existing hospitals into county or some other territorial groups. With a key hospital in the centre, and special or smaller institutions in relation thereto, beds can be economised, and treatment, so to speak, distributed; every patient will be somewhere in the circle, and will have a ready means of access to the centre or other point most suitable to his or her needs.

The moral of it all is that once again the voluntary system has shown a power of adaptation, a resource native to itself, which makes the suggestion of State interference simply irrelevant. Those who want it deserve to have it; that is all. The appeal and the survey together show a capacity in the voluntary system which assures it of success. The system which can produce such a scheme will not fail to produce the money for it. We have no doubt that the response will be a triumph.

